# Delivering project SCORE in competitive youth sport settings

**DOI:** 10.3389/fspor.2024.1439822

**Published:** 2024-08-20

**Authors:** Marta Ferreira, Fernando Santos, María A. Fernández-Villarino, Jason Mergler, Leisha Strachan, Dany J. MacDonald

**Affiliations:** ^1^ Escola Superior de Educação, Instituto Politécnico do Porto, Porto, Portugal; ^2^ Facultad de Ciencias de la Educación y del Deporte, Universidad de Vigo, Vigo, Spain; ^3^ inED, Centro de Investigação e Inovação em Educação, Escola Superior de Educação, Instituto Politécnico do Porto, Porto, Portugal; ^4^ Faculty of Kinesiology and Recreation Management, University of Manitoba, Winnipeg, MB, Canada; ^5^ Department of Applied Human Sciences, University of Prince Edward Island, Charlottetown, PE, Canada

**Keywords:** coach training, coaching, athlete development, youth development, values, competitive sports

## Abstract

**Introduction:**

This study evaluates the effectiveness of the Project SCORE intervention in fostering Positive Youth Development (PYD) within competitive youth sport settings in Portugal. Project SCORE is an online PYD-focused tool developed to assist coaches in promoting the 4Cs—competence, confidence, connection, and character—within their coaching.

**Methods:**

The research involved 13 coaches and 70 youth athletes from football and rowing teams. Methodologically, this study analyzed the pre- and post- Project SCORE intervention data, assessing the perceptions of coaches and athletes towards the development of the 4Cs.

**Results:**

Results indicated significant improvements in athletes' perceptions of 4Cs post-intervention, and among coaches' perceptions, there was a significant improvement in the practice and transfer of life skills. Particularly, coaches showed enhanced abilities in fostering life skills and facilitating the transfer of these skills to competitive environments, although some dimensions like sport climate did not sustain positive changes.

**Discussion:**

The findings highlight the benefits of customized PYD-based programs in competitive youth sports and suggest the need for further research to enhance their widespread and consistent implementation.

## 1 Introduction

For Positive youth development (PYD) through sport has been used as an approach to enhance sporting experiences and enable young people to become active participants in society (1). PYD deviates from a perspective that views youth as a problem to be solved, an incomplete project unable to satisfy social demands. Instead, focuses on youth's strengths and aims to develop individuals' assets (2). Concerning PYD through sport, some of the most common outcomes that may come as a result of sport-based PYD programming are life skills and the 4Cs (3–5). Participation in sport-based PYD programs has been associated with the development of life skills in youth athletes (6).

Examples of life skills are respect, leadership, and teamwork that can be used in areas outside sport (7, 8). To encourage coaches to foster life skills in sport programs, Bean et al. (7) proposed a life skills implicit-explicit continuum. This continuum highlights how coaches can use a range of strategies, both implicit (e.g., creating a positive climate, building meaningful relationships with athletes) and explicit (e.g., directly teaching life skills and incorporating them into practice). Holt et al. (4) hypothesised that a PYD climate together with an explicit life skill focus may generate better PYD outcomes than a PYD climate alone, suggesting both implicit and explicit strategies have value in coaching practice. On one hand, implicit strategies have proved useful in fostering PYD in certain sports and cultures (9). On the other hand, explicit strategies that include setting life skills as an objective for practice and providing opportunities to develop these skills (e.g., providing a leadership role) have been considered meaningful (10).

With regards to the 4Cs (11, 12), this framework includes four outcomes that should be attained in sport-based PYD programs: (a) competence, (b) confidence, (c) connection, and (d) character. First, competence refers to social, cognitive and motor skill development. Second, confidence connects to feelings of self-worth and self-efficacy. Third, connection is associated to the quality of the relationships established between different actors within the youth sport system (e.g., the coach and athletes). Finally, character represents the ability to respect for rules and norms, as well as present prosocial behaviours towards others. These 4Cs serve as outcomes of quality sport-based PYD programming (12, 13). Taken together, approaches focused on teaching valuable skills can provide valuable characteristics for the development of youth in and outside sport environments and guide sport-based programming (14).

Based on the benefits that a PYD approach can offer and that coaches are one of the most influential actors that impact youth development, various efforts have been made by researchers aiming to assist and educate coaches (15–17). To equip coaches with the necessary skills and competencies to foster PYD and life skills, several efforts have been made to develop PYD-focused coaching education programs (CEPs) [e.g., (10, 18, 19)]. MacDonald and colleagues (18) studied the impact of a PYD-focused CEP on coach behaviours and athlete perceptions within competitive youth sport. The study found that coaches in the intervention group exhibited an increase in PYD-oriented behaviours during the intervention, but these changes were not sustained at the follow-up phase throughout the latter portion of the season. On the other hand, athletes' perceptions of coach PYD behaviour did not show significant changes over time. Although the results suggest that PYD-focused CEPs may influence coach behaviour in the short term, more research is needed to understand how to maintain these changes and foster positive athlete developmental experiences across all types of youth sport programs (recreational and competitive). Other studies have also supported significant changes in variables such as the quality of coach-athlete relationships and life skills teaching as a consequence of PYD-focused CEPs (20, 21).

One PYD-focused CEP that has been used in past research is Project SCORE (19). It's an online tool developed in 2011 by Canadian researchers that aims to assist coaches in fostering PYD (22). Strachan and colleagues (19) developed Project SCORE with the theoretical foundation of the 4C's. Initially, Project SCORE was a self-administered online resource with asynchronous video content. However, researchers have emphasized the need for contextual adaptations based on participants' learning needs, available time, and priority given to PYD (22, 23). Learner-centred approaches have been considered useful in fostering meaningful coach learning (24). To better understand how learner-centred approaches may apply to coach education, according to Paquette and Trudel (25) has pointed to the need to (a) use contents that instigate engagement with practice; (b) ensure a safe and supporting learning environment that respects coaches' needs; (c) define learning outcomes based on coaches’ wants and needs; (d) create solid grounds for coaches to become autonomous while searching for knowledge; (e) consider power dynamics and social pressures; and (f) see coach improvements as a pathways to search for novelty and more contextualized knowledge. Hence, Project SCORE may be paired with other approaches towards coach learning and complementary strategies [see (23) for an example].

Conversely, there are some studies that report the ineffectiveness of PYD-focused CEPs. For instance, Camiré and colleagues (10) evaluated the effectiveness of the “Coaching for Life Skills” online training program for high school coaches. For the most part, these coaches were involved in competitive programs. Results showed no significant changes in variables such as coach-athlete relationships. This may be the case due to the fact that competitive youth sport programs place substantial pressures for coaches to increase performance outcomes, which may come at the cost of other developmental outcomes such as life skills. Furthermore, other studies that have examined the effectiveness of PYD-focused CEPs have reached similar findings [e.g., (26)]. MacDonald et al. (18) suggest that PYD-focused CEPs may influence coach behaviour in the short term, although more research is needed to understand how to maintain these changes and foster positive athlete developmental experiences across all types of youth sport programs (recreational and competitive). Therefore, the evidence available on the effectiveness of PYD-focused CEPs targeting coaches in competitive environments is still unclear, which supports the need for more studies associated to this line of inquiry.

Competitive youth sport programs create a complex setting to establish PYD due to the reward system in place and pressures to perform (27, 28). Moving forward, there is the need to examine how PYD-focused CEPs such as Project SCORE can be tailored to fit different socio-cultural contexts and coaching contexts such as competitive youth sport programs. Furthermore, longitudinal designs are needed to analyse how coaches and athletes change their perceptions because of PYD-focused CEPs as well as other environmental factors and pressures (e.g., period of the sport season). Therefore, the present study aims to analyze the impact of Project SCORE on coaches and youth athletes' experiences within two competitive youth sport settings during a sport season.

## 2 Methods

### 2.1 Participants

The final number of participants consisted of 13 coaches and 70 athletes. The coaches (7 males, 6 females) were on average 26.2 years old (SD = 7.7). The athletes (15 males, 55 females) were on average 14.1 years old (SD = 2.2). In order to recruit participants, local sport administrators were contacted to share study details within their organization. Potential participants could participate based on the following criteria: (a) they were involved in a local competitive youth sport organization; (b) youth sport participants were between 10 and 17 years old (29); and (c) coaches were interested in using sport as a context for PYD.

The recruiting process resulted in 15 coaches and 81 athletes from football (soccer) and rowing teams. Both sport organizations were involved in competitive contexts and participated in provincial and national level competitions. Although 15 coaches started the study protocol, two coaches left their position during the study process. Out of the 81 athletes, 11 were removed from the analysis because they did not complete the entirety of the data collection. It should be noted that one of the sport organizations who delivered competitive football programs had female teams that were involved in the intervention, which explains the disparity between male and female athletes.

### 2.2 Materials

#### 2.2.1 Athletes measures

The data was collected using the measures contained within the 4 Cs toolkit (12, 13) to assess athletes' perceptions of connection, confidence, competence, and character.

##### 2.2.1.1 Connection

Connections between coaches and athletes were measured through the Coach-Athlete Relationship Questionnaire [CART-Q; (30)], which consists of an 11-item questionnaire that assesses the dimensions of closeness, commitment, and complementarity using a seven-point Likert scale (1 - strongly disagree and to 7 - strongly agree). Cronbach alpha values were .58 pre intervention and .67 post intervention for commitment, .73 pre and .86 post for closeness and 65 pre and .76 post for the complementarity.

##### 2.2.1.2 Confidence

Confidence was measured with the Trait Robustness of Self-Confidence for Athletes [TROSCI; (31)], which is a seven-item questionnaire that evaluates two dimensions (i.e., robust self-confidence and unstable self-confidence) through a nine-point Likert scale (1 - strongly disagree and to 9 - strongly agree). The Cronbach alpha values were .77 (pre) and .79 (post) for robust self-confidence and .83 (pre) and .86 (post) for unstable self-confidence.

##### 2.2.1.3 Competence

Competence was measured with the Sport-Confidence Inventory [SCI; (32)], a 14-item questionnaire that evaluates three dimensions using a seven-point Likert scale (1 - absolutely not at all and to 7 - totally certain): confidence in physical skills and training; confidence in cognitive efficiency; and confidence in resilience. The Cronbach alpha values for the measure's subscales ranged from .80 to .87 pre intervention and from .73 to .91 post intervention.

##### 2.2.1.4 Character

The Prosocial and Antisocial Behaviour in Sport Scale [PABSS; (33)] is a 20-item questionnaire that assesses the four dimensions of (i) antisocial behaviour towards teammates, (ii) antisocial behaviour towards opponents of the other team, (iii) prosocial behaviour towards teammates, and (iv) prosocial behaviour towards opponents of the other team. This scale uses a five-points Likert scale (1 - never and to 5 - almost always). The Cronbach alpha values for the measure subscales were between .58 and .82 at pre-intervention and between .47 and .69 post-intervention.

#### 2.2.2 Coaches measures

Coaching for life skills. For coach participants, the Portuguese Coaching Life Skills in Sport Questionnaire [P-CLSS-Q; (34)] was used. The scale is a 30-item questionnaire which assesses the five factors of: (i) structuring and facilitating a positive climate in sport, (ii) discussing life skills, (iii) practicing life skills, (iv) discussing the transfer of life skills, and (v) practicing life skills transfer. The scale uses a six-point Likert scale (1 - strongly disagree and to 6 - totally agree) to assess life skills teaching. In the present study, Cronbach alpha values for the measure's subscales were between .80 and .95 at pre-intervention and between .70 and .93 post-intervention.

### 2.3 Procedure

#### 2.3.1 Prior to data collection

The study was approved as part of a larger project by the ethics committee of a local polytechnic higher education institution (Polytechnic Institute of Santarém, number 072021). Project SCORE (19) was utilized as a resource to help youth sport coaches understand how to infuse the 4Cs in their coaching practice. The following sections describe (i) the pre-intervention phase (initial data collection with coaches and athletes); (ii) implementation of Project SCORE (description of the training); and (iii) the follow-up phase (post intervention data collection).

#### 2.3.2 Pre-intervention phase

Once participants were recruited, coaches and athletes completed the outlined questionnaires before the Project SCORE workshop. For coaches, it took 10–15 min, and for athletes, 15–20 min to fulfil the questionnaires. The questionnaires were completed before or after a team practice, with the primary researcher present to clarify any questions.

#### 2.3.3 Project SCORE implementation

The implementation of Project SCORE involved a co-developed CEP based on a learner-centered approach [e.g., (24)]. In the present study, Project SCORE was delivered by the primary researcher following a learner-centered approach. This ensured a genuine concern towards learners, their needs, and how meaningful learning occurs (35).

The Project SCORE workshop was used to create an environment conducive to meaningful learning and aimed to forge a positive relationship between the coach developer and coaches. The workshop consisted of a two-hour, in-person, CEP. During the workshop, several topics concerning each of the 4Cs were addressed. Therefore, some strategies concerning the 4Cs were presented such as creating opportunities for athletes to plan and lead the warm-up and prompt a discussion with athletes about life skills transfer. In addition to the primary material, additional topics (e.g., generation Z athletes, parent engagement) which emerged organically were discussed. The workshop took place in January 2023 and the coaches implemented the material with their athletes from February to May 2023. Therefore, the implementation process occurred during the competitive period of the season (approximately halfway through their season).

During the implementation phase of Project SCORE content, the primary researcher (who served as the coach developer) was available to coaches to discuss planning, potential strategies, observe practices, and provide feedback. A total of 71 practices were observed throughout the 21-week period of January 2023 and May 2023. Informal meetings between the first author and each coach took place at least once a week depending on their needs (*n* = 57). Coaches could choose to meet either individually or in groups (39 individual meetings and 18 group meetings were held). Each meeting lasted between 30 and 150 min and occurred before and/or after practices. The informal meetings aimed at helping coaches understand how to plan and implement Project SCORE strategies, envision novel approaches, and consider athletes' developmental needs in a meaningful manner.

During these meetings the coach developer aimed to instigate coach reflection rather than provide answers and recommendations. Indeed, the coach developer used prompts to promote meaningful discussions such as what were the main objectives this week?, how have the athletes responded to the strategies you applied?, what were the main difficulties you had?; and what can you improve in your intervention?. A group messaging tool was also created for coaches within each youth sport organization to facilitate the exchange of discussion, sharing ideas, and reflexive routines. It should be noted that coaches were not pressured to implement the strategies explored in Project SCORE, but instead encouraged to use this resource as a starting point to promote PYD.

#### 2.3.4 The follow-up phase

After the Project SCORE implementation phase ended, the measures described in the pre-intervention stage were used. This phase occurred between May 2023 and June 2023 throughout a three-week period.

### 2.4 Data analysis

Firstly, an examination of data normality was conducted, revealing that the variables did not follow a normal distribution (*p* > .05). For this purpose, the Kolmogorov-Smirnov test was employed due to the sample size being greater than 50. Gender differences were not calculated in this analysis. In order to control for individual variability in different moments, a mixed linear model (MLM) for repeated measures was chosen. This approach is suitable for analyzing data when observations are not independent, which is often the case in sports science research involving repeated measures on the same subjects or clustered data such as teams (36). This MLM accommodates data that may not meet the normal distribution assumption and can handle complex data structures with fixed (e.g., moment and sport) and random (e.g., participant's ID) effects (37).

Accordingly, this model provides the significant differences between factors using the Bonferroni *post hoc* test. The Cohen's d effect size (ES) was provided as a quantitative measure of the magnitude of the difference or relationship, offering insights beyond mere statistical significance, and helping to understand the practical significance of the findings. It was interpreted via the following ranges: <0.20 = trivial effects, 0.20–0.49 = small effects; 0.50–0.70 = moderate effects, and >0.8 = large effects (38).

## 3 Results

Table 1 provides the results across the subscales of the athlete questionnaires and the results of the analyses investigating differences pre- and post- intervention.

**Table 1:** Mean and standard deviations scores of pre and post intervention across the subscales of athlete questionnaires.

	M Pre (SD)	M Post (SD)	*p*	E.S.	*α*
Connection					
Commitment^b^	5.9 (.97)	6.5 (.74)	<.001	.57	.58–.67
Closeness^b^	6.8 (.50)	6.9 (.41)	.132	.18	.73–.86
Complementarity^b^	6.5 (.56)	6.8 (.52)	.009	.31	.65–.76
Confidence					
Robust^c^	5.6 (1.9)	5.5 (2.1)	.988	.01	.77–.79
Unstable^c^	5.6 (2.1)	4.2 (2.4)	<.001	.56	.83–.86
Competence					
Skills^b^	6.0 (.92)	6.4 (.68)	<.001	.51	.85–.83
Cognitive^b^	5.8 (.94)	6.3 (.64)	<.001	.60	.80–.73
Resilience^b^	5.5 (1.1)	5.9 (1.1)	.011	.36	.87–.91
Character					
Anti-Teammates^a^	1.6 (.60)	1.4 (.39)	<.001	.42	.77–.67
Anti-Opponents^a^	1.7 (.62)	1.6 (.39)	.154	.20	.82–.69
Pro-Teammates^a^	3.7 (.83)	3.9 (.73)	.020	.30	.66–.58
Pro-Opponents^a^	2.3 (.94)	2.6 (.83)	.025	.26	.58–.47

^a^ 1–5 Likert-scale.

^b^ 1–7 Likert-scale.

^c^ 1–9 Likert-scale.

### 3.1 Athletes' perceptions

#### 3.1.1 Connection

Differences between pre- and post- assessment were analyzed on the CART-Q. For the coach-athlete relationships, statistically significant increases were identified for commitment (*p* < .001) and complementarity (*p* = .009). The PYD-focused CEP demonstrated to have a moderate effect size from .01–.57 in some subscales, which highlights areas where the program was particularly effective.

#### 3.1.2 Confidence

Regarding self-confidence, the results from the unstable self-confidence subscale showed a significant change with unstable confidence decreasing at follow-up (*p* < .001). The effect of CEP had a moderate size effect (ES = .56).

#### 3.1.3 Competence

The three subscales of the competence measure showed statistically significant increases at follow-up. Specifically, skills, cognitive, and resilience subscales of the measure positively increased. In addition, effect sizes across subscales were .51 for skills, .60 for cognitive, and .36 for resilience which represent small to moderate effects of the Project SCORE CEP.

#### 3.1.4 Character

For the character scale, statistically significant differences were found in the subscales of: Anti-Teammates (AT; *p* < .001), Pro-Teammates (PT; *p* = .02), and Pro-Opponents (PO; *p* = .025). In general, the effect of the CEP was small (.30–.42).

### 3.2 Coaches' perceptions

Coaching for life skills. Across the subscales of the measure, four showed increases following the workshop while one (sport climate) decreased (Table 2). The decrease in sport climate was significant at follow-up (*p* = .042) while practicing life skills (*p* = .039) and practicing life skills transfer (*p* = .005) increased significantly. On the other hand, discussing life skills and the discussing of life skills transfer did not change significantly. Regarding effect sizes of the CEP, values ranged between .24 and .98 indicating small to large effects over the study (see Table 2).

**Table 2:** Pre and post scores of coach participants along with pre- and post- difference, effect sizes, and pre- and post- reliability analyses.

	M Pre (SD)	M Post (SD)	*p*	E.S.	α
Sport climate	5.7 (.36)	5.44 (.37)	.042	.63	.83–.85
Discussing ls	4.9 (.99)	5.05 (.74)	.602	.24	.91–.90
Practicing ls	3.7 (1.5)	4.67 (.99)	.039	.64	.95–.88
Discussing transfer ls	4.5 (1.1)	4.90 (.89)	.266	.32	.82–.93
Practicing transfer ls	3.0 (1.2)	4.25 (1.01)	.005	.98	.80–.70

ls, life skills.

## 4 Discussion

The purpose of the present study was to assess the effectiveness of a Project SCORE intervention on coaches' and athletes' perceptions involved in competitive youth sport programs. In addition, this is one of the first studies to evaluate the impact of a CEP using Project SCORE as the base for knowledge (19, 23). Another unique aspect is the fact that studying youth from a Portuguese competitive sport system provided contextualized insights about if/how Project SCORE can impact the development of the 4Cs in athletes and coaching for life skills in coaches. Studying athletes within a competitive context was deemed necessary to grow our understanding of PYD within contexts that are often deemed as inconsistent with the PYD framework and holistic development [e.g., (39)].

Traditionally, there have been perceived conflicts between competitive contexts and psychosocial development that can lead to misconceptions about the development of psychosocial skills in competitive youth sport (40). Therefore, the present study provides an opportunity to reflect on how PYD and athletic performance can be positioned as interrelated and inseparable objectives. Indeed, past research has shown cases of tensions between competition, performance and PYD [e.g., (18)]. However, findings of the present study highlight how Project SCORE had a positive effect on both athletes’ and coaches' perceptions within a performance context and provide opportunities for policy makers, sport organizations, coaches, and CEPs to consider personal development within competitive contexts.

In the present study, there were significant differences between pre- and post-measures concerning athletes' perceptions associated with the 4Cs. With regards to connections, particularly those between coaches and athletes, there were significant differences on the quality of the coach-athlete relationship for both to complementary and commitment. Past research has highlighted the importance of the coach-athlete relationship in achieving PYD outcomes such as life skills [e.g., (4, 41)] and mediating intentions to continue sport participation (42). Despite the lack of significant results concerning closeness, it is important to note that athlete scores were high (i.e., the maximum was seven points and the athletes scored 6.9 after the PYD-focused CEP).

Hence, to interpret these findings in a comprehensive manner there is the need to consider several factors. Athletes reported high perceptions towards the coach-athlete relationship at the pre-intervention phase which may help explain why (a) there were no significant differences on closeness and (b) a ceiling effect may have occurred at the follow-up phase. Considering the competitive nature of the Portuguese youth sport system (27), coaches may have increased their ability to create relationships through meaningful and long-lasting cooperation (i.e., complementary and commitment) to achieve better performance outcomes. In this sense, coaches may see value in a PYD mandate, particularly in improving the quality of coach-athlete relationships because these are a key component of the coaching process and may help achieve performance outcomes. Additionally, PYD can be a part of a set of demands imposed by sport organizations, which occurred in the present study. It should be noted that coach and athlete participants were recruited from two sport organizations who were willing to consider PYD and partake in this intervention. In other words, sport administrators were the ones deciding whether Project SCORE would be implemented, but coaches also played a complementary role in this decision-making process. Subsequently, coaches may have valued the presence of a coach developer because they wanted to optimize professional development processes, become more effective, as well as fulfill organizational demands.

Previous studies grounded on partnerships and collaborative efforts between researchers and practitioners have demonstrated positive outcomes (10), indicating that such cooperations are crucial. This may suggest the need to carefully consider how to establish meaningful partnerships with sport organizations and develop contextualized strategies [e.g., (10, 43)]. For instance, in some cases PYD as an organizational objective may need to be mandated by sport administrators and policy makers that hold social capital over coaches and other actors. Such mandates could continue to reinforce the notion that PYD and performance can indeed be complementary rather than incongruent when considering holistic youth development. As research on PYD continues to develop and expand to multiple countries across the globe (44), Portugal may reflect an example of how PYD and performance are starting to become interrelated components of youth development [e.g., (28)].

Findings showed significant differences between pre- and post-measures. Specifically, there were significant decreases in unstable self-confidence, despite no significant increases in robust self-confidence. Concerning competence, there were significant changes in skill, cognitive development, and resilience. Lastly, with regards to character there were decreases in antisocial behaviours with teammates and increases in prosocial behaviours with teammates and opponents. Taken together, these findings support previous studies of PYD-focused CEPs which have positively impacted athletes' perceptions of the 4Cs (20, 21). One important consideration is that the Project SCORE workshop was embedded by coaches as part of a competitive context and once again demonstrates that these contexts are not incongruent with the promotion of PYD. These findings further demystify the common conceptualizations that competitive environments are independent from PYD mandates (40).

Our findings of positive impacts of a PYD-focused CEP with youth sport participants engaged in highly competitive environments adds to our understanding of personal development within youth sport. Previous studies have reported more limited impacts of PYD-focused CEPs on athletes' perceptions [e.g., (26, 45)]. For instance, Junior et al. (45) highlighted how competitiveness may influence coaches' perception of PYD and effectively connect this approach to performance objectives. The findings of the current study support Junior's et al. (45) recommendation that coaches and parents should not only create an environment that develops life skills but also discourage antisocial behaviors towards opponents. Nonetheless, competitive environments may have athletes disregard behaviours towards opponents and/or neglect the need to change certain behaviours in competitions.

To further understand the interrelatedness of competitive sport and positive development it is important to delve deeper into the context. At different times over the course of a competitive season, PYD and performance will vary in importance. For example, athletes and coaches may value PYD more readily during less competitive parts of a season or during practice while focusing on performance during specific competitions or tournaments. This suggests a non-linear process where PYD and performance are continuously valued at different moments. These fluctuations can lead to both implicit and explicit approaches to PYD (4) if the participants and coaches agree that PYD is a valuable goal in youth sport, but it is addressed more at certain times and in specific contexts.

As mentioned above, coach participants in the current study were conveniently selected and were interested in pursuing PYD within their context. The positive outcomes in the promotion of PYD post-workshop corroborates previous studies (43, 46). Despite the positive outcomes, coaches decreased in the perceived ability to promote a positive sport climate and discussing life skills and life skills transfer. These findings may showcase context-specific variables associated with this socio-cultural context that resonate across cultures. First, Bean et al. (7) highlighted how implicit-explicit strategies related to coaching life skills should be positioned as interrelated. In other words, coaches may use a variety of implicit-explicit strategies at the same time and value diverse types of strategies differently across time. One should have in mind that explicit strategies may be positioned as critical for performance development [see (39)]. Second, the fact there were no significant changes on coaches' perceived ability to discuss life skills and life skills transfer may be explained by the cultural relevance of PYD in coaches’ discourses. In other words, PYD and life skills are North American concepts (47) and may not be part of coaches' discourses, language and interactions. These concepts may be transformed into more culturally appropriate and common words and expressions such as “values”, “attitudes” and “personal development” can enhance understanding and acceptance among coaches, enabling them to integrate these ideas more effectively into their practices and interactions with athletes. Third, coaches may have been concerned about athletes' responses to these new concepts in such a performance-oriented environment and losing credibility. Explicit discussions around PYD may not be seen as automatically useful and/or needed. These results may also have derived from the fact that a learner-centered approach was used, which enabled the coach developer to tailor Project SCORE per coach participants' learning needs, as well as leverage PYD with performance objectives. Finally, the design of the intervention, especially the sustained engagement between the coach developer and the coaches over a 21-week period played a crucial role in effectively infusing implicit and explicit strategies into coaching practice. This prolonged interaction allowed the coach developer to tailor contents, strategies and approaches to the unique contexts of each coach, thereby enhancing the overall impact of the intervention. It was important to ensure the PYD-focused CEP was aligned with the demands of competitive youth sport programs.

In the present study, there are some limitations and future directions that need to be considered moving forward. First, the study involved a small sample of coaches and athletes, which creates challenges in extrapolating the findings to similar settings. Second, due to wide age range used for the athlete's sample (e.g., 10–17 years old), combined with the small sample, was not possible to evaluate the differences regarding the age of the athletes. Third, some of the Cronbach's Alpha values were below usually acceptable criteria due to the sample size. Finally, the athlete sample was composed of mostly female athletes, which may also limit generalizability. Moreover, sample characteristics did not enable an examination of differences between genders amongst coaches and athletes. Together, these limitations encourage us to interpret the findings with caution but also open up avenues for further research to better understand how additional variables influence coaching practice.

Based on these limitations, future studies could attempt to refine our understanding of PYD within competitive contexts. There is the need to assess interrelationships between competitive climates and personal development. In most cases, PYD has been assessed and investigated detached from other coaching concerns, objectives and practices (e.g., physical development, tactical development). Essentially, if the purpose is for coaches to coach holistically and satisfy competitive demands, future research designs may need to follow the same approach. On this notion, with regards to the evaluation of the effectiveness of PYD-focused CEPs such as Project SCORE efforts may need to be deployed towards understanding coach and athlete perceptions and behaviours in competitions, moving beyond simply analyzing practices. Also, it is important to address potential differences in coach-athlete relationships based on gender and age as these could influence the dynamics and outcomes of coaching practice. There is the need to explore how the relationship between coaches and athletes varies according to age and gender. Finally, to appropriately map change across youth sport systems, future research endeavours may also need to investigate the evolution between personal and performance development within competitive youth sport programs.

## 5 Conclusions

Within Portuguese youth sport, there has historically been an emphasis on competition (27). Despite past research in the context not demonstrating overly positive effects of a PYD-focused CEP (18), the current study found positive effects with competitive coaches and athletes. The exact mechanisms for these results are unknown and would warrant further research. However, given that researchers in Portugal have been promoting PYD for the better part of the last decade (18, 48), it is possible that the current findings represent small changes in openness and behaviour toward PYD. Once again, further research needs to evaluate this claim but ultimately, the findings hint towards possible integration of PYD within performance-based contexts. As such, coaches, coach developers, sport administrators, and policy makers can use the present findings in evaluating their programs and adjusting them to provide space for PYD to live alongside performance.

## Data Availability

The original contributions presented in the study are included in the article/Supplementary Material, further inquiries can be directed to the corresponding author.
